# An Absolute Calibration of the National Bureau of Standards Thermal Neutron Flux

**DOI:** 10.6028/jres.067A.019

**Published:** 1963-06-01

**Authors:** E. J. Axton

## Abstract

The NBS Thermal Neutron Flux has been calibrated in terms of the gold thermal neutron capture cross section. The effective thermal neutron (below cadmium cutoff energy) flux density is estimated to be 4307 ± 2 percent *n*/cm^2^ sec in September 1961. This figure is in agreement with a recent value quoted by the NBS.

## 1. Introduction

The NBS Standard Thermal Neutron Flux has been calibrated in terms of the *B*(*n, α*) cross section [De Juren and Rosenwasser, 1954] and of the gold thermal neutron capture cross section [Mosburg and Murphey, 1961]. The present calibration, based on absolute counting of ^198^Au activity by the 4*πβ‒γ* coincidence method, represents part of a program of international comparisons of thermal neutron flux density measurement.

## 2. Method and Procedure

The method is similar to that used for the absolute calibration of the neutron flux in the AERE reactor GLEEP [Axton, 1963]. This calibration was based on absolute counting of ^56^Mn and of ^198^Au by the 4*πβ–γ* coincidence method, and some of the same gold foils and the same counting equipment are used in the present experiment, so that the various correction factors associated with the foils and equipment have already been evaluated and, where appropriate, are reproduced in table 1.

A pair of foils (*G*_1_ *G*_2_) 30 mg/cm^2^ in thickness and 1 cm diam were irradiated in the flux for one week. A similar pair (*G*_3_, *G*_4_), encased in cadmium 0.1 cm thick, were irradiated for 11 days.

The foils were counted in the 4*πβ* counter of the coincidence equipment previously described [Axton, 1963]. For *G*_1_ *G*_2_, the total counting rate was approximately 11 c/s against a background of 0.9 c/s. For *G*_3_, *G*_4_, the counting rate was approximately 2.2 c/s, against a background counting rate of 0.9 c/s. In view of the low counting rate it was not possible to make coincidence measurements on these foils. However, the ratio between *N_β_*, the net counting rate in the 4*πβ* counter, and *N*, the absolute disintegration rate has been measured for these foils a number of times both before and after this irradiation in the course of the GLEEP flux measurement.

The reaction rate *D*_0_ per milligram of foil is obtained from the equation

$$D_{0}=\frac{D\;\exp[\lambda (t_{3}-t_{2})]\lambda (t_{4}-t_{3})}{\left\{1-\exp[-\lambda (t_{2}-t_{1})]\}\{1-\exp[-\lambda (t_{4}-t_{3})]\right\}}$$ (1)

where λ is the decay constant of ^198^Au and *D* is the average disintegration rate per mg from time *t*_3_ to time *t*_4_ of a foil irradiated from time *t*_1_ to time *t*_2_.

The thermal neutron flux density, defined as *n*_th_ *v*_0_, where *n*_th_ is the neutron density below the cadmium cutoff energy, and *v*_0_=2200 m/sec, is given by

$$n_{\text{th}}v_{0}=\frac{(1/F)(D_{0}-D_{0}{\;}_{\text{Cd}}F_{\text{Cd}})A}{0.001\;N_{0}g\sigma_{0}}$$ (2)

where

*D*_0 cd_: is the reaction rate per mg obtained with the foil under cadmium.
*F*_Cd_: is a correction factor for the attenuation of resonance neutrons in the cadmium.
1/*F*: is a correction factor for thermal neutron flux density depression and self shielding.
*gσ*_0_: is the effective absorption cross section for neutrons with a Maxwellian distribution of velocities at 20 °C.
*σ*_0_: =98.8b (Hughes and Schwartz, 1958).
*g*: =1.0053 (Westcott, 1960).
*A*: =Atomic Weight.
*N*_0_: =Avogradro’s Number.

The values of both *F* and *g* are dependent upon the temperature *T* associated with the most probable velocity in the Maxwellian distribution. The temperature is assumed here to be *T*_0_ to conform to the value of *g* quoted by Mosburg and Murphey [1961]. *T*_0_=293.6 °K and *kT*_0_ is the energy corresponding to the neutron velocity *v*_0_=2200 m/sec at which thermal cross sections are normally tabulated.

1/F=X/[12−E3(X)]whereX=Σefft=N0tA×1.08gσ0(T0/T)1/2

and

$$E_{3}(X)=\int_{1}^{\infty }u^{-3}\exp\;(-uX)du$$

*t* is the foil thickness in g/cm^2^.

## 3. Results

The results are presented in table 2 from which it can be deduced that the effective thermal neutron (below cadmium cutoff energy) flux density is given by

$$n_{\text{th}}l_{0}=4307\;n/{\text{cm}}^{2}\;\text{see}.$$

For comparison purposes the values of *n*_th_*v*_0_ obtained at NBS are presented in table 3, column 1, and corrected to September 1961 in column 2.

However, the procedure used to calculate the neutron flux differs from that of Mosburg and Murphey [1961] in three respects.

Firstly, the value 1.048 calculated by Mosburg and Murphey, and used by them for the correction factor *F_C_*_d_ is believed to be too large. The calculation of this factor is difficult, since it involves an effective cross section for cadmium. To obtain such an effective cross section it is not sufficient to calculate the absorption of the resonance neutrons in the cadmium; nor is it sufficient to measure the reduction of the activity produced by a further equal thickness of cadmium. This follows because the activity of the cadmium covered foil is not produced wholly by the resonance-energy neutrons. Mosburg and Murphey used the cadmium total cross section at the gold resonance energy in their calculation of *F*_Cd_. However, to satisfy the experimentally derived value for *F*_Cd_ quoted in table 1, it is necessary to use only the capture cross section plus a fraction of the scatter cross section. This fraction is expected to vary with foil thickness as will the relative contribution of the resonance- energy neutrons to the activity of the cadmium covered foil. However, there are insufficient data available at the present time to establish variations of *F*_Cd_ with foil thickness. Thus, the experimentally determined value for these particular foils and cadmium covers (table 1) is preferred. If the results of Mosburg and Murphey, who used similar thicknesses, are recalculated using this value of F_Cd_, the flux value is raised by about 0.6 percent.

Secondly, the effective cross section *gσ*_0_ is used instead of *σ*_0_ to derive the neutron flux from the foil activity. This results in a reduction of about 0.5 percent and almost cancels out the first change.

Thirdly, the effective cross section 1.08 *gσ*_0_ is used instead of *σ*_0_ to derive the foil self-absorption correction.

The results of Mosburg and Murphey 1961] have been recalculated with these changes to produce the revised value for the neutron flux density shown in column 3.

$$\text{Thus}\;\text{the}\;\text{ratio}\frac{\text{NPL}\;\text{flux}\;\text{density}\;\text{measurment}}{\text{NPL}\;\text{flux}\;\text{density}\;\text{measurment}}\text{is}\;1.020,$$

which reduces to 1.018 if the adjustments to *F*_Cd_ and to the cross sections are made in the calculation of the NBS gold value.

It is sometimes of interest to specify the neutron flux density in terms of the total neutron density *n*, or of the density in some specific component of the flux. In order to do this with detectors whose cross section does not follow the 1/*v* law, it is necessary to assume a spectrum shape, which, in the case of well- moderated reactors, usually comprises a Maxwellian distribution of velocities at temperature *T* °K and a *dE/E* component terminated at an effective lower limit *μkT*. With the present system of point sources, it is unlikely that the spectrum follows the 1/*E* law very closely. Nevertheless, in the absence of specific information, the *dE/E* assumption has been made for the purposes of the following calculations, and the results therefore should be treated with reserve.

The relative intensity of the *dE/E* component is derived from cadmium ratio measurements. The cadmium ratio *R*_Cd_ (ratio of activity obtained with bare foil to that obtained with cadmium covered foil) is given by the equation

$$R_{\text{Cd}}=\frac{F{\left(r\sqrt{T/T_{0}}\right)}^{-1}+G_{t}s_{0}/g}{1/K+f(\delta )G_{t}(s_{0}/g-W)}$$ (3)

which is based on the equation of Walker et al. [1960] with (a) the addition of the self-shielding factor *F*, and (b) the assumption of zero penetration of thermal neutrons through the cadmium.

*s*_0_: is a function of the resonance integral *s*_0_=17.3 [Westcott 1960] (*s*_0_ is the *T=T*_0_ value of Westcott’s *s*_4_).
*W*: is a function of the resonance integral below the cadmium cutoff energy. *W*=0.027. [Walker et al. 1960].
*K*: is a function of the cadmium cutoff energy. For 0.1 cm Cd, *K*=2.2931 [Westcott et al. 1958].
*r*: is a measure of the relative intensity of the *dE/E* component. The fraction of the neutron density in the *dE/E* component of flux is $4r{\left(\pi \mu \right)}^{-\frac{1}{2}}.$
*g*: 1.0053.
*G_t_*: accounts for the self-screening of the resonance-energy neutrons in the foil.
*f*(*δ*): accounts for the attenuation of the resonance-energy neutrons in the cadmium cover.
*G_t_* and *f*(*δ*): are reproduced in table 1 from a previous paper [Axton, 1963].

For a full discussion on the meanings of these terms the reader is referred to the literature.

Thus, with the experimentally determined value for *R*_Cd_ the value of *r* can be calculated from eq (3). The value so obtained is *r*=0.0212. The total flux density *nv*_0_ is then given by

$$\begin{array}{l} nv_{0}=n_{\text{th}}v_{0}(R/R-1)=n_{\text{th}}v_{0}{\left(1-r/K\right)}^{-1}\\ \;=n_{\text{th}}v_{0}/0.9908=4347\;m/{\text{cm}}^{2}\;\text{see} \end{array}$$

where *R* is the cadmium ratio which would be obtained with a thin 1/*v* detector. *R=K/r* from eq (3) with *F*=1, *T=T*_0_, *s*_0_=*W*=0. It appears that changes as high as 20 percent in the value of *r* have very little effect on the value obtained for the total neutron flux density.

The Maxwellian flux density *n_m_v*_0_ (where *n_m_* is the neutron density in the Maxwellian component of the flux) is given by

$$n_{m}v_{0}=nv_{0}\left(1-\frac{4r}{{\left(\pi \mu \right)}^{\frac{1}{2}}}\right)=0.9751\;nv_{0}=4239\;m/{\text{cm}}^{2}\;\sec.$$

The value of *μ*, which is determined by the effective lower energy limit of the *dE/E* component of the flux, is a matter for some discussion. The value used here (3.681) is that for which *s*_0_ was computed.

## 4. Accuracy of Results

Table 4 shows the various errors *α* associated with the calculation of the thermal neutron flux density, together with the weights *ω* with which they influence the result.

The sum [Σ(*ωα*)^2^]^1/2^ is less than ±1 percent. In addition, there may be a systematic error not exceeding ±1 percent associated with the absolute measurement of the radioactivity. The combined error is thus ±2 percent.

It is concluded that NBS and NPL measurements of thermal neutron flux density are in agreement within the limits of experimental error.

## Figures and Tables

**Table 1:** 

Foil	Mass mg	Nominal thickness mg/cm^2^	Foil data					
			*N_β_/N*	1/*F*	*F*_Cd_	*f*_(δ)_	*G_t_*	*gσ*_0_
								
*G*_1_	23.19	30	0.6841	} 1.028	1.01	0.991	0.441	99.32
*G*_2_	23.48	30	.6924					
*G*_3_	23.44	30	.6896					
*G*_4_	22.97	30	.6961					

**Table 2:** Results

Foil	*N_β_* c/s corrected for decay	*D*_0_ dps/mg	*D*_0_ Cd dps/mg	*R*_Cd_	*r*	*n*_th_ *v*_0_ September 1961
						
*G*_1_	19.961	1.507	……	……	……	……
*G*_2_	20.209	1.489	……	……	……	……
*G*_3_	3.4257	……	0.2260	……	……	……
*G*_4_	3.2981	……	.2200	……	……	……
Mean	………	1.498	.2230	6.72	0.0212	4307

**Table 3:** Comparison of NBS and NPL measurements

	*n*_th_*v*_0_		
	Published value *n*/cm^2^ sec	Corrected for polonium growth September 1961 *n/*cm^2^ sec	Corrected for change in *F*_Cd_ and in effective cross sections September 1961 *n/*cm^2^ sec
			
NBS (Gold) Mosburg-Murphey, [1961]	4167±1.5%	4188±1.5%	4195
NBS (Boron) De Juren and Rosenwasser, [1954]	4276±2%	4297±2%	4297
Weighted mean^a^	4203±1.5%	4224±1.5%	4229±1.5%
Ratio NPL measurement/NBS weighted mean	………	1.020	1.018

a The means given here are those quoted by Mosburg and Murphey (1961), who give arbitrary weights of 2 and 1 to the gold and boron measurements, respectively.

**Table 4:** Errors associated with flux density measurement

Error	Weight *ω*	Error *α*
		
Counting statistics:		%
(Bare foil and background)	0.832	0.4
(Foil in Cd and background)	.124	1.4
*N_β_/N* (Bare foil)	.832	.75
*N_β_/N* (Foil in Cd)	.124	.75
Mass (Bare foil)	.832	.1
Mass (Foil in Cd)	.124	.1
*F*	1	.3
*F*_Cd_	.175	.5
*gσ*_0_	1	.4
